# Critical care physicians treating COVID-19: mind the nervous system!

**DOI:** 10.1186/s13054-020-03026-x

**Published:** 2020-06-08

**Authors:** Julian Bösel

**Affiliations:** 1 Department of Neurology, Kassel General Hospital, Mönchebergstr. 41-43, 34125 Kassel, Germany; 2 Department of Neurology, Heidelberg University Hospital, Heidelberg, Germany

Dear Editor,

Critical care for severely afflicted patients with COVID-19 is certainly heralded by pulmonary failure. However, growing evidence indicates that the virus causing COVID-19 may directly or indirectly affect the nervous system, especially in ICU-dependent cases.

Some patients may present with symptoms suggesting neurologic involvement such as loss of smell or taste, headaches, dizziness, confusion, obtundation, seizures, or focal neurologic deficits. Others may not display those hints on admission, but still develop neurologic manifestations. A recent review in *Critical Care* by Kotfis et al. has highlighted ICU-delirium as an association with the latter, but the spectrum is likely broader.

As examples of direct neurologic affection by SARS-CoV-2, meningoencephalitis in a Japanese patient [1] and fatal encephalitis in a US patient [2] have been published, along with virus detection in the cerebrospinal fluid or in neurons/endothelial cells at autopsy, respectively.

Rather indirect consequences of COVID-19 may be forms of encephalopathy, reflected by agitation, confusion, or delirium, possibly following cytokine storms [3]. Cerebrovascular events, ischemic or hemorrhagic, even in young patients without vascular risk factors [4] may be caused by blood constellations of inflammation and hypercoagulability. Another para-infectious example is the inflammatory polyneuropathy Guillain-Barré syndrome [5] (Table 1).

**Table 1:** Selection of ICU-relevant publications on neurologic manifestations of COVID-19

First author	Location	Study type	Sample	Main outcomes
Helms	Strasbourg, France	Retro study	58	ICU patients, median age 63 y, assessed during wake up trial or after cessation of sedation: CNS symptoms in 84%, of these agitation in 69%, corticospinal tract affections in 67%, neurocognititive dysfunction after discharge in 36%. Diagnostics: MRI in 11 of 13 with hypoperfusion, ischemic stroke (3), or leptomeningeal contrast enhancement; CSF in 7 with elevated protein, no SARS-CoV-2 detection
Moriguchi	Yamanshi, Japan	Case report	1	24 yo patient with headache, fever, seizures and obtundation, meningoencephalitis; MRI with hyperintensities, CSF with SARS-CoV-2 detection
Paniz-Mondolfi	New York City, USA	Case report	1	74 yo patient with fever and confusion, died after severe ICU course; on autopsy detection of SARS-CoV-2 in neurons and endothelial cells of frontal brain
Toscano	Brescia/ Pavia/ Alessandria, Italy	Case series	5	23–77 yo patients, 3 ventilated, with tetraplegia 5–10 days after COVID-19 symptoms, typical signs of Guillain -Barré syndrome on elecrophysiology tests and in CSF without SARS-CoV-2 detection
Oxley	New York City, USA	Case series	5	Patients < 50 y, sudden and severe neurologic deficits from large vessel occlusion despite absence of stroke risk factors; laboratory constellation of inflammation and hypercoagulability

*Abbreviations*: *COVID-19* coronavirus disease 2019, *y* year(s), *yo* year-old, *CSF* cerebrospinal fluid, *SARS-CoV-2* severe acute respiratory distress syndrome–coronavirus 2, *ICU* intensive care unit

Pathophysiologically, the routes of neuroinvasion may be trans-synaptically, e.g., via cranial nerves connecting the (naso) pharynx and pulmonary organs with the central nervous system. Another very likely route is via the blood and endothelial cells at the blood-brain barrier. Also, lymphatic and enteric ports of entry are being discussed. Thereafter, cell entry of the virus works via ACE2, which is expressed by not only cells of the respiratory tract, but also endothelial cells, neurons, and glia.

At present, the rate and relevance of neurologic affection in COVID-19 critically ill patients are unclear, with studies on neurologic aspects just being started. However, these first observations should alarm intensivists all over the world, as additional damage to the brain and nerves will further impact on prognosis. Some supporting therapies may even end up detrimental if these comorbidities go unnoticed. But the latter will invariably happen if those are not actively looked for. Hence, intensivists of all disciplines should employ a low threshold of suspicion for nervous system involvement when treating COVID-19 in the ICU. Neurologic consults, neurocritical care expertise, brain imaging, lumbar puncture, and brain autopsy may all be warranted more often than we think.

Yours sincerely,

Julian Bösel

FNCS, FESO, President-elect German Society for Neurologic Intensive Care and Emergency Medicine (DGNI)

## Data Availability

No own data, all relevant publications cited.
